# Change in Japanese children’s 24-hour movement guidelines and mental health during the COVID-19 pandemic

**DOI:** 10.1038/s41598-021-01803-4

**Published:** 2021-11-26

**Authors:** Kim Hyunshik, Ma Jiameng, Lee Sunkyoung, Gu Ying

**Affiliations:** 1 Faculty of Sports Education, Sendai University, 2-2-18 Funaokaminami, Shibata-machi, Miyagi-ken, 9891693 Japan; 2 Department of Life Physical Education, Myongji University, 34 Geobukgol-ro, Seodaemun-gu, Seoul, 03674 Republic of Korea; 3 College of Sports Science, Shenyang Normal University, No. 253, Huanghe North Street, Huanggu District, Shenyang City, Liaoning Province China

**Keywords:** Health care, Medical research

## Abstract

Specialized guidelines are required for the health behaviors of vulnerable populations such as children. This is especially true during the COVID-19 pandemic, wherein major lifestyle changes have occurred, especially among young children. The present study aims to use longitudinal data to understand changes in the physical activity, screen time, sleep, and mental health of preschoolers in Japan during the COVID-19 pandemic, compared to pre-pandemic period. Subjective and objective measures were used to assess the variables of interest longitudinally. It was found that physical activity, adherence to WHO-recommended screen time, and prosocial behaviors decreased significantly. On the other hand, sedentary time and hyperactivity increased. Our results are consistent with findings from other countries. The implications with respect to outdoor playtime, screen-time in the context of online learning during the pandemic, and the effects of parents’ mental health on preschool-aged children are discussed.

## Introduction

The Coronavirus disease (COVID-19) first emerged in 2019. With its rapid spread, COVID-19 was not localized to China and spread to Japan, Korea, the United States, Spain, Italy, and France in early 2020^1^. The World Health Organization (WHO) declared the COVID-19 outbreak a pandemic on March 11, 2020^2^. China, where the first known cases of COVID-19 were identified, has set up a strict blockade, surveillance of the case, and temporary shelters. Governments of many countries implemented strategies such as closed borders and social distancing to minimize the spread of COVID-19 and protect their citizens^3^. After Japan confirmed its first case on January 14, 2020, a state of emergency was declared in major cities on April 16, 2020, due to the rapid spread of COVID-19^4^. Additionally, restrictive measures were enforced, including temporary closure of elementary, junior high, and high schools across the nation, social distancing (2 m), restricted access to local communities, social gatherings, sports centers, playgrounds, and public parks. Protecting physical and mental health during a highly restrictive situation is essential, especially for vulnerable populations such as children.

In 2019, the WHO published global guidelines on physical activity (PA), sedentary behavior (SB), and sleep for preschool-aged children^5^. It is essential to increase their level of PA, reduce SB, and ensure sufficient sleep within the 24-h cycle to maintain and promote the optimal health of preschool-aged children. It has been reported that behaviors associated with movement, including PA, SB, and sleep, contribute to optimal physical and mental health among preschool-aged children by strengthening their immune systems^6–8^. However, COVID-19 has changed the lifestyles of children, families, and local communities. Since the pandemic began, preschool-aged children have been less likely to engage in organized physical or outdoor activities and are more likely to spend more time indoors. Consequently, there is an increase in unhealthy behaviors such as SB based on screen time among preschool-aged children^9,10^. Globally, preschool-aged children are locked in their homes and have not been away from their friends for so long before. The current social crisis provoked by COVID-19 has had a substantial impact on the daily movements and behaviors of preschool-aged children. Previous studies reported that during the COVID-19 pandemic, only 17% of Chinese children engaged in adequate PA, with 66% being physically inactive^9^; similarly, Canadian children also had reduced PA^10^. Moreover, a study on the impact of lockdown on children during COVID-19 reported that children spent their free time watching television (TV) or using the Internet instead of engaging in PA, confirming this dramatic change^11,12^.

The WHO reported that in addition to the physical problems affecting children, lockdowns and social distancing measures have increased people’s anxiety and caused stress, triggering psychological and mental health problems^13^. Approximately 10–20% of children and adolescents worldwide suffer from mental disorders such as depression, anxiety, and aggressive behavior^14^. Further, a recently published WHO 24-h movement guideline (24-h MG) has provided evidence supporting the association of mental health with PA^15,16^, screen time^17,18^, and sleep^19,20^. The present study differs from previous studies in that it used one-year longitudinal data from pre-and during-COVID-19 and objective means to examine WHO 24-h MG and changes in mental health.

Thus, this study aimed to examine the changes in PA, screen time, sleep, and mental health of preschool-aged children during the COVID-19 pandemic compared to the pre-pandemic period.

## Methods

### Study design and participants

This longitudinal study collected data for a year: pre-and during-COVID-19. A convenience sample from seven childcare centers in northeastern Japan that volunteered to participate in the “Study on the Improvement of Life Habits among Children in East Asia” was selected in consideration of the study’s purpose.

**Figure 1:**
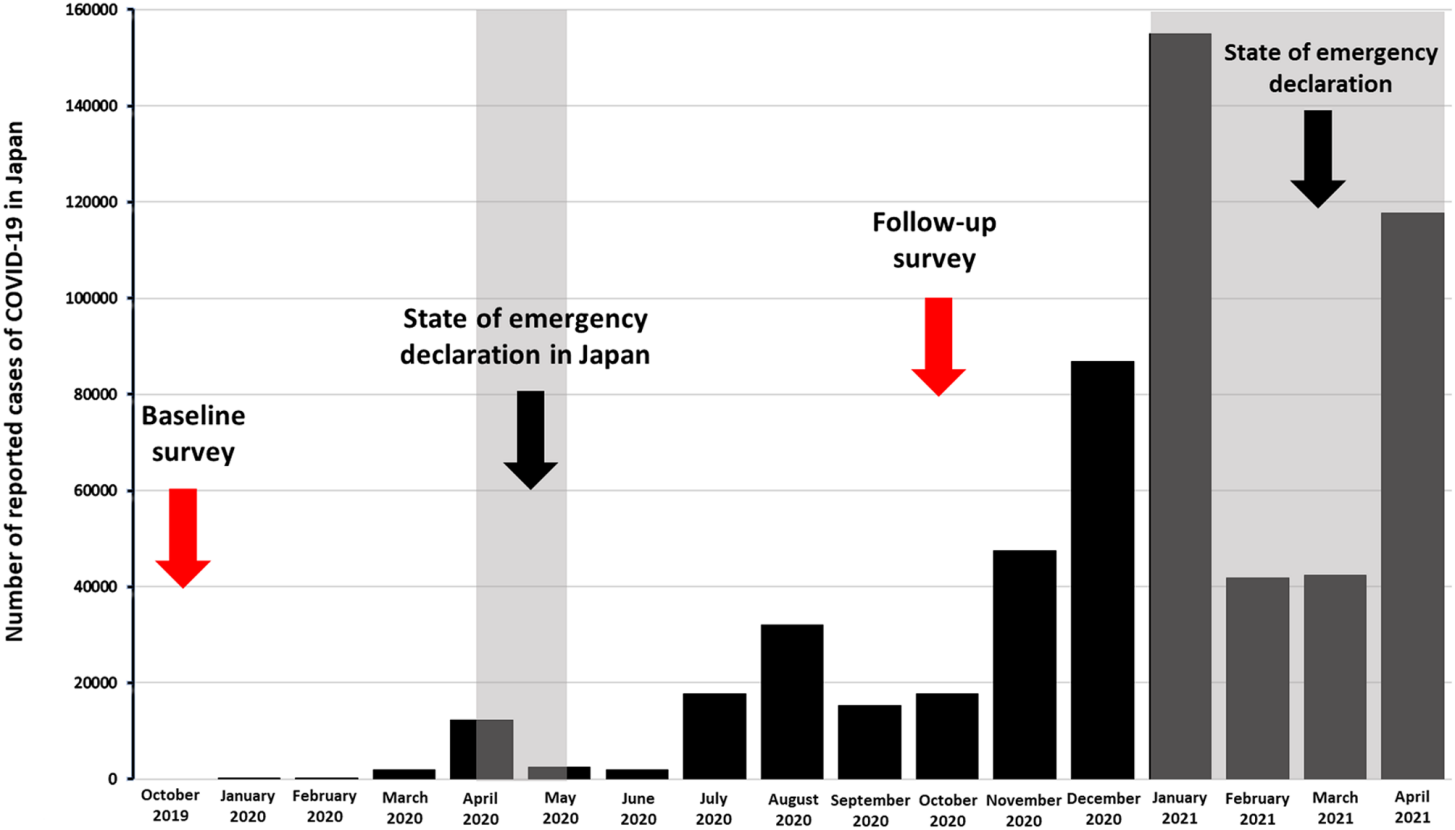
Research period and number of reported cases of COVID-19 in Japan.

Of the preschool-aged children attending one of seven childcare centers in the Miyagi Prefecture in the northeastern region of Japan (n = 920), young children aged 0–2 years (n = 416) were excluded, and only children aged 3–5 years (n = 504) were enrolled in the study. The first accelerometer measurement and questionnaire were administered in October 2019 (as shown in Fig. 1). After the measurement and survey, data from five-year-old children not included in the longitudinal data (n = 157), data of children withdrawn from the study due to withdrawal of consent (n = 14), the criterion for accelerometer measurement was data from devices not being worn for more than 600 min a day and more than four days a week (n = 23), and erroneous data with missing responses in the questionnaire (n = 8) were excluded. This resulted in a total of 301 participants (boys: 51.9%; girls: 48.1%) that were included in the analysis at T1 (2019 survey). At T2 (2020 survey was conducted using the same method as the 2019 survey), the criterion for accelerometer measurement was data from devices not being worn for more than 600 min a day and more than four days a week (n = 10), and erroneous data with missing responses in the questionnaire (n = 1) were excluded from the T1 (n = 301) data. The remaining data (n = 290) were analyzed comparatively.

The participants of this study were healthy children aged 3–5 years without any physical or mental disabilities. Before the study, we provided a study information sheet and consent form to the children’s caregivers, and data from those who signed the consent form were included in the analysis. All participants and their parents provided written informed consent before the beginning of the study according to the guidelines of the Declaration of Helsinki. Additionally, we ensured that all experiments were performed in accordance with relevant guidelines and regulations. The study received prior approval from the Sendai University Ethics Committee, Faculty of Sports Science (SU29-22).

### Measurements

#### Physical activity

The amount of PA, a triaxial accelerometer (Active Style Pro HJA-750C, Omron Health Care Co., Ltd., Kyoto, Japan) was used. The Active Style Pro provides the metabolic equivalent of task (MET) values derived from predictive equations for adults; however, it has been reported that MET values result in overestimated results for children compared to adults^21,22^. Therefore, we used the following conversion Eqs. (1) and (2) obtained from the results of previous studies:

1 $${\text{Ambulatory activities:}} \, 0.{6237}\, \times \,{\text{the value of MET by the Active style Pro}}\, + \,0.{2411}$$

2 $${\text{Non-}} {\text{ambulatory activities:}} \, 0.{6145}\, \times \,{\text{the value of MET by the Active style Pro}}\, + \,0.{5573}$$

The participants were asked to wear an accelerometer on their waist for one week from when they woke up until they went to sleep (7:00 and 21:00), except when taking a shower or swimming. If the accelerometer value remained at 0 for 20 min or longer, then it was assumed that the participant was not wearing the accelerometer. In terms of PA, the triaxial accelerometer measured sedentary time (≤ 1.5 metabolic equivalents (METs)), light-intensity (1.6–2.9METs), and moderate and vigorous PA (3 METs or above); and these measures were evaluated every 10 s. The data were extracted to measure PA per day, when the participants wore the accelerometer for 600 min or more per day, for four days per week^23^. Weekly average moderate-to-vigorous PA (MVPA) was calculated as: weekly average MVPA = ((weekday MVPA) + (weekend MVPA)/2).

#### Screen time

Screen time refers to the time spent on screen-based behaviors, including recreational, stationary, sedentary, and active screen time^23^. Screen time was assessed by asking parents how much time their children spent watching TV/videos and using smartphones/tablets in the past week using the following questions: (1) How much time on average does your child spend on a day watching TV or videos? (2) For how long, on average in a day does your child use electronic devices, such as smartphones, tablets, and computers? Subsequently, parents were asked to indicate the average number of days per week, and weekends their child spent on-screen-viewing time based on six options: 0, 1–29, 30–59, 60–119, 120–179, or ≥ 180 min. Parents also provided the daily average screen time on weekends and weekdays in written answers^25^. We calculate the average time spent on the screen-viewing activities per week by multiplying the assessed screen-viewing time to the mid-category values of the duration of the activity per day. Subsequently, the child’s average daily screen time was calculated (average daily screen time = (weekday screen time × 5) + (weekend screen time × 2) /7).

#### Sleep duration

Sleep duration was measured using the questions, “How many hours does your child sleep at night?” and “How long is your child’s nap time?” as answered by parents. Daily sleep duration was calculated as follows: ((sleep duration + nap time)/2).

#### Strengths and difficulties questionnaire (SDQ)

The SDQ is a questionnaire used to assess psychopathology, positive strengths, and behavioral problems in children aged 3–16 years, and it can be efficiently completed by a parent or teacher^26^. Owing to its user-friendliness, the SDQ has been translated into more than 75 languages^27^, and several versions that meet the needs of researchers, clinicians, and educators are currently being used^28^. The SDQ is divided into 25 items consisting of five subscales: emotional, conduct, hyperactivity, peer problems, and prosocial skills, which can be scored based on a Likert scale (each scale consists of five items). When added together, the first four symptoms would yield Total Difficulties scores (TDS), which are based on 20 items. The Japanese version of the SDQ used in this study was confirmed to have high reliability (α = 0.81)^29^ and validity^30^.

#### Adherence to the 24-h movement guidelines (WHO 24-h MG)

The following recommendations were used to evaluate the new WHO 24-h MG for preschool children^5^: PA guidelines (180 min of total PA including 60 min/day of moderate to vigorous PA), screen time guidelines (less than an hour per day), and sleep duration guidelines (10–13 h within 24 h).

#### Demographic variables

A questionnaire was used to survey the participants’ demographical variables. Information on the children’s sex, age, bedtime, waking time, weight, and height was obtained from their parents. The height and weight of preschool children were measured in units of 0.1 cm and 0.1 kg, respectively, and objective measurements were made by the researchers. The BMI z-score was calculated according to the WHO growth criteria^31^. For participants that were five years and below, overweight and obesity were classified as BMI z-score above 2 standard deviations and 3 standard deviations, respectively^32^.

### Statistical analysis

The participant characteristics were coded as continuous variables (sex, age, bedtime, wake-up time, height, body weight, and Z-score); therefore, they were analyzed with t-tests, and the mean values were compared between genders. Changes in the study parameters were examined by comparing pre-and during-COVID-19 data. PA and sedentary time from the WHO 24-h MG were assessed based on accelerometer data, and screen time and sleep duration were analyzed with paired sample t-tests to examine the important changes in the data obtained from the questionnaire. These results indicate the changes in the average time and satisfaction rates for the parameters in the WHO 24-h MG pre-and during-COVID-19. Furthermore, the average changes in the total SDQ score and scores of the five subscales (emotional symptoms, conduct problems, hyperactivity-inattention, peer relationship problems, and prosocial behaviors) were examined. The percentages of preschool-aged children who complied with the WHO 24-h MG recommendation pre-and during-COVID-19 were compared using a Wilcoxon signed-rank test. For the level of statistical significance, p-values were set to < 0.05. Data analyses were performed using IBM SPSS version 26.0 (IBM, Armonk, NY, USA).

## Results

Table 1 shows the descriptive statistics of the preschool-aged children who participated in this study before and a year after the COVID-19 outbreak. The mean age was 3.6 ± 0.3 years before COVID-19 and 4.8 ± 0.3 years during COVID-19, and a significant association was observed (p < 0.001). Height (pre-COVID-19: 101.0 ± 5.7 cm, during-COVID-19: 108.4 ± 5.8 cm) and body weight (pre-COVID-19: 15.9 ± 2.1 kg, during-COVID-19: 18.6 ± 2.8 kg) were also significantly associated (p < 0.001).

**Table 1:** Descriptive characteristics of participants.

	Pre-Covid-19		During-Covid-19		*p-Value*
	n = 301 (2019 sample)		n = 290 (2020 sample)		
Sex (girls)	153	47.1%	153	47.8%	
Age (years)	3.6 ± 0.3		4.8 ± 0.3		** < 0.001**
Bedtime (minute)	1285.2 ± 38.0		1287.9 ± 37.4		0.541
Waketime (minute)	407.3 ± 36.1		410.0 ± 34.7		0.298
Weight (kg)	15.9 ± 2.1		18.6 ± 2.8		** < 0.001**
Height (cm)	101.0 ± 5.7		108.4 ± 5.8		** < 0.001**
BMI z-score	0.14 ± 0.8		0.18 ± 0.8		0.471

Bold values indicate a factor of significant relevance.

Table 2 compares the PA, SB, and sleep between the pre-and during-COVID-19 periods. Weekday moderate to vigorous physical activity (MVPA), measured using an accelerometer, significantly decreased from 93 to 88 min. Moreover, while weekday light PA (479 min to 450 min) and weekend light PA (488 min to 436 min) decreased, weekday sedentary time (164 min to 174 min) and weekend sedentary time (175 min to 197 min) increased significantly.

**Table 2:** Change of physical activity, sedentary behavior, sleep in pre- and during COVID-19 pandemic.

	Pre-Covid-19 n = 301	During-Covid-19 n = 290	Mean change (95% CI)	*t*	*p-Value*
	Mean (SD)	Mean (SD)			
**Physical activity (min/day)**					
MVPA (weekdays)	93.0 (27.3)	88.0 (26.2)	5.0 (0.1, 9.9)	2.005	**0.046**
MVPA (weekend)	83.4 (34.1)	77.9 (29.7)	5.5 (− 13.1, 2.0)	1.454	0.148
LPA (weekdays)	479.5 (86.2)	450.6 (89.4)	28.9 (12.8, 44.9)	3.542	** < 0.001**
LPA (weekend)	488.3 (113.9)	436.4 (102.2)	51.9 (71.9, 26.8)	4.089	** < 0.001**
Steps (weekdays)	8857.5 (2388.1)	8547.5 (2606.8)	310.0 (− 141.6, 761.6)	1.353	0.177
Steps (weekend)	7488.2 (3019.9)	7267.2 (3005.1)	221.0 (− 480.0, 922.9)	0.623	0.535
**Sedentary behavior (min/day)**					
Sedentary time (weekdays)	164.4 (49.7)	174.3 (49.5)	− 9.9 ('− 0.6, '− 19.2)	− 2.100	**0.037**
Sedentary time (weekend)	175.8 (62.8)	197.6 (66.9)	− 21.8 (− 37.9, − 5.7)	− 2.675	**0.008**
TV/DVD (weekdays)	96.9 (63.0)	98.7 (63.0)	− 1.8 (− 9.2, 12.7)	− 0.322	0.748
TV/DVD (weekend)	163.1 (93.9)	165.1 (96.3)	− 2.0 (− 14.7, 18.8)	− 0.241	0.810
Smartphone/tablet (weekdays)	31.5 (44.7)	34.9 (29.4)	− 3.5 (− 11.9, 4.9)	− 0.821	0.412
Smartphone/tablet (weekend)	51.4 (65.7)	61.2 (4.5)	− 10.0 (− 21.9, 1.9)	− 1.662	0.098
**Sleep (min/day)**					
Sleep duration	619.1 (37.9)	623.8 (39.6)	− 4.7 (− 1.6, 11.1)	− 1.463	0.145

Bold values indicate a factor of significant relevance.

*SD* standard deviation, *MVPA* moderate to vigorous physical activity, *LPA* light physical activity, *PA* physical activity

Table 3 shows the changes in the WHO 24-h MG adherence rate for each movement and the combinations between the pre-and during-COVID-19 periods. The adherence rate for each factor and combination decreased during COVID-19 compared to pre-COVID-19 rates, but the change was insignificant. However, the rate of screen time adherence significantly decreased (27% to 19%).

**Table 3:** Proportion of WHO 24-h MG recommendations and combinations in pre-and during COVID-19 pandemic.

Meeting recommendations	Pre-Covid-19 n = 301		During-Covid-19 n = 290		Mean change	*p*-Value
	N	%	N	%		
None	20	6.6	30	10.3	− 3.7	0.101
Physical activity	255	84.7	239	82.4	2.3	0.593
Screen time	79	27.2	60	19.9	7.3	**0.010**
Sleep duration	252	83.7	233	79.2	4.5	0.437
Physical activity + screen time	64	22.1	58	19.3	2.8	0.679
Physical activity + sleep duration	190	63.3	182	62.8	0.5	0.831
Screen time + sleep duration	64	21.3	60	20.7	0.6	0.792
Physical activity + screen time + sleep duration	47	15.6	39	13.4	2.2	0.323

Bold value indicates a factor of significant relevance.

Meeting therecommendations is defined as 180 min of total PA including more than 60 min/day of moderate-to-vigorous PA, no more than 60 min/day for screen time, and between 10 and 13 h/day for sleep duration. PA was based on accelerometer-determined while and sleep duration and screen time were self-reported.

**Figure 2:**
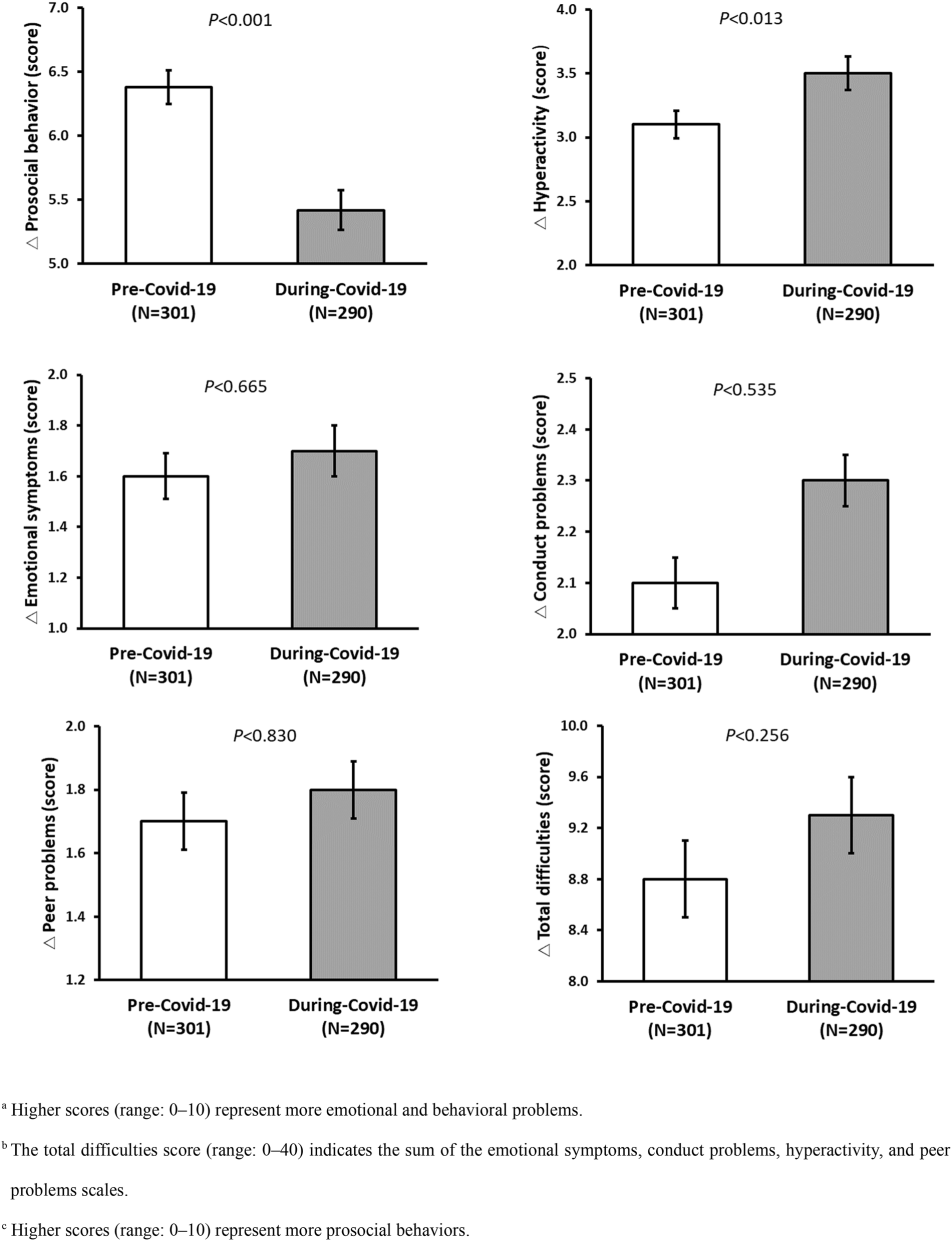
Comparison of SDQ score due to differences in pre-and during COVID-19 pandemic.

We compare children’s mental health pre-and during-COVID-19 with the changes in the total SDQ score, and scores of each of the five subscales (emotional symptoms, conduct problems, hyperactivity-inattention, peer relationship problems, and prosocial behaviors) were compared between the two periods (Fig. 2). The mean score for the prosocial behavior subscale decreased during the COVID-19 pandemic compared to the pre-COVID-19 score (6.4 to 5.4; p < 0.001). Furthermore, the mean score for the hyperactivity subscale increased during the COVID-19 pandemic compared to the pre-COVID-19 score (3.1 to 3.5; p < 0.013).

## Discussion

This longitudinal study aimed to investigate the effects of the COVID-19 pandemic on preschool-aged children’s PA, screen time, sleep, and mental health using subjective and objective measures.

Our results demonstrated that compared to the pre-COVID-19 period, weekday MVPA, weekday light PA (LPA), and weekend LPA decreased. In contrast, weekday and weekend SB increased as measured using an accelerometer during the COVID-19 pandemic. Our results on PA were consistent with the results of a Spanish study^33^ and a Chilean study^34^ on preschool-aged children, and with the results of Tunisian^35^, Chinese^9^, Canadian^10^, US^11^, and German^36^ studies on school-aged children between 5 and 17 years of age with different restrictions such as lockdowns. In particular, one notable finding of this study is that LPA and MVPA were affected by COVID-19 and that the levels during weekends and weekdays decreased.

Children generally engage in PA, participating in organized play, games, and dance in institutions (childcare, kindergarten, and elementary school) and spending time in the playground and park after school^37^. However, the transition to online learning and social restrictions from the COVID-19 pandemic hindered preschool-aged children’s participation in school-based or community-based PA through physical education, sports, or other activities. Our results demonstrate that weekday MVPA decreased after the COVID-19 outbreak, suggesting that social restrictions also reduced organized PA among children in childcare centers (wearing a face mask during exercise, bans on group play, and restricted play areas). It has been reported that children and adolescents are spending less time outdoors during the COVID-19 pandemic^10,11^. Reduced outdoor playtime may have a substantial impact on reduced MVPA and LPA among children. A systematic review reported that the guidelines for maintaining an active lifestyle during the COVID-19 pandemic published by many international organizations have little consideration of vulnerable groups (older adults and young children) and that even during this period, individuals need to engage in periodic PA to maintain good mental and physical health^38^. As demonstrated in the present study, outdoor play is crucial to boost PA during the COVID-19 pandemic, and policies that enable children to play outdoors with minimal infection risk (such as the use of a face mask and physical distancing in playgrounds, parks, and other green areas) should be devised to address this issue. Both weekday and weekend SB, objectively measured using an accelerometer, increased during the COVID-19 pandemic. Our results are consistent with a previous Spanish study on preschool-aged children, reporting that PA decreased and SB increased^33^. The WHO 24-h MG suggests that PA, SB, and sleep are mutually influential through physiological interactions throughout 24 h. Further, the time spent on one activity may affect another activity. Therefore, each activity should not be treated independently^8^.

Subsequently, we compared the rate of adherence to the screen time recommendation of the WHO 24-h MG pre-and during-COVID-19, and the rate significantly decreased from 27.2% before the pandemic to 19.9% after the pandemic. This is markedly low even compared to Asian countries with similar cultures before the pandemic, such as Japan^22^, China^39^, and Singapore^40^. After the COVID-19 outbreak, social distancing measures and parents’ self-implemented restrictions on their children due to the risk of infection led to dramatically less outdoor time and increased screen time at home. Previous studies also reported that screen times have significantly increased since the implementation of COVID-19-related quarantine measures^9,37,41^. Screen time is an integral social phenomenon in modern society, as screen-based online learning enhances children’s self-esteem, social skills, and knowledge^42^. However, the focus should be on reducing screen time on activities, which may have an adverse impact on recreational time, such as playing games and watching TV. Adherence to screen time recommendations is more strongly associated with family factors, such as parents’ screen time modeling, their TV watching, and house rules, as opposed to individual or social factors^43^. Hence, subsequent intervention studies should consider parents’ behaviors and support. Moreover, educational and psychological support for children during the current virtual era of education must focus on those who may experience higher levels of anxiety during the pandemic^44^.

Our results also demonstrate that restricted social activities due to COVID-19 had a considerably negative impact on preschool-aged children’s mental health. These results were consistent with findings from other countries, where administrative measures such as lockdowns imposed due to COVID-19 had adverse impacts^45–47^. Further, a study on Japanese elementary school students reported increased externalizing problems, such as hyperactivity and inattention^48^. Our results were also consistent with the results of population-based studies conducted before the COVID-19 pandemic, wherein boys demonstrated more hyperactivity and inattention than girls (boys: 3.2, girls: 2.5)^49^. Of the various mental health problems affecting preschool-aged children, externalizing problems (hyperactivity and inattention) are associated with the individual, family, and social factors^50^. Hence, COVID-19-triggered anxiety and depression among parents may have contributed to children’s externalizing problems^51^. Since the social restrictions imposed in response to the COVID-19 pandemic have led to increased family time, during which families build stronger relationships with one another, children with hyperactivity and inattention would face a high level of family conflict. The low level of prosocial behaviors, such as cooperativity and empathy, may also be attributable to fewer opportunities to interact with other people outside the childcare center due to the COVID-19 pandemic. Parents, childcare teachers, educators, and administrators must take action to reduce the mental health repercussions of COVID-19 in preschool-aged children. Programs promoting mental health and prevention tailored toward young children in need of care must be developed to prepare ourselves for a potential second wave of COVID-19 or the post-COVID-19 era.

Some limitations need to be considered in the interpretation of the findings of this study. First, since this study was targeted at preschool children in the northeastern region of Japan, it is not clear whether it can be generalized nationally to preschool children in Japan. Second, the study employed a questionnaire survey wherein the parents or family members of the child observed the child’s screen time and sleep duration and filled out the questionnaire, as in other studies with preschool children. Third, as parents’ psychological condition has been reported to influence their evaluation of their children’s behaviors^52^, mental health and emotional symptoms are likely to be underestimated. These factors should be considered when interpreting the results^53^.

## Conclusion

Compared to the pre-COVID-19 period, preschool-aged children engaged in less weekday MPVA and weekday and weekend LPA but engaged in more screen time during the COVID-19 pandemic. The rates of adherence to each recommendation and its combinations in the WHO 24-h MG declined overall, with a significant decrease in the rate of adherence to the screen time recommendation. With respect to mental health, the COVID-19 pandemic has had an adverse impact on prosocial behavior and hyperactivity. These results emphasize the need to implement strategies that increase PA, reduce SB, including screen time, and ensure adequate sleep duration among preschool-aged children during the COVID-19 pandemic to prevent long-term health risks.

## Data Availability

Data provided in this study are available upon request by the corresponding author. The data were not made public because basic information on children was de-signed to be tested.
